# Prediction of viscosity behavior in oxide glass materials using cation fingerprints with artificial neural networks

**DOI:** 10.1080/14686996.2020.1786856

**Published:** 2020-07-22

**Authors:** Jaekyun Hwang, Yuta Tanaka, Seiichiro Ishino, Satoshi Watanabe

**Affiliations:** a Department of Materials Engineering, The University of Tokyo, Tokyo, Japan; b Department of Physics, The University of Tokyo, Tokyo, Japan; c Institute of Industrial Science, The University of Tokyo, Tokyo, Japan; d Center for Materials Research by Information Integration, Research and Services Division of Materials Data and Integrated System, National Institute for Materials Science, Tsukuba, Japan

**Keywords:** Chemical composition, machine learning, materials informatics, neural network, oxide, glass, viscosity, 60 New topics/Others, 107 Glass and ceramic materials, 404 Materials informatics/Genomics

## Abstract

We propose a novel descriptor of materials, named ‘cation fingerprints’, based on the chemical formula or concentrations of raw materials and their respective properties. To test its performance, this method was used to predict the viscosity of glass materials using the experimental database INTERGLAD. Using artificial neural network models, we succeeded in predicting the temperature required for glass to have a specific viscosity within a root-mean-square error of 33.0°C. We were also able to evaluate the effect of particular target raw materials using a model trained without including the specific target raw material. The results show that cation fingerprints with a neural network model can predict some unseen combinations of raw materials. In addition, we propose a method for estimating the prediction accuracy by calculating cosine similarity of the input features of the material which we want to predict.

## Graphical abstract

**Figure:**
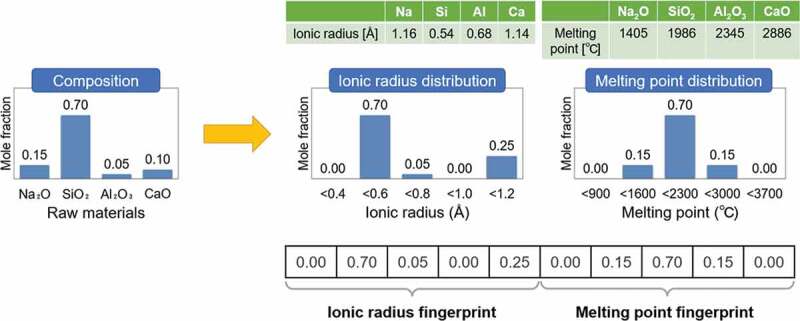

## 1. Introduction

A glass is an amorphous solid-state material obtained from rapid cooling from the high-temperature liquid state to a temperature below the glass transition temperature ($T_{g}$). The rapid cooling suppresses crystallization and causes a supercooled liquid state. The supercooled liquid becomes more viscous as the temperature is lowered. Understanding the behavior of its viscosity η is important in the case of glass materials, and many studies have been conducted to obtain this [1–6].

One of the important aspects of viscosity in the behavior of glass is its temperature dependence. To examine this behavior, the Angell plot is often used [4], in which the logarithm of η versus the inverse of temperature T multiplied by the glass transition temperature ($T_{g}/T$) is plotted. The Angell plots for various glass materials can be classified into three types, namely, the ‘strong,’ ‘fragile,’ and ‘intermediate’ plots. The strong plot is almost a straight line, whereas the fragile plot is a convex curve. The intermediate type is in the middle of the strong and fragile plots. In the cases of the strong and intermediate types, which include most of the geochemical and metallic glasses, the Angell plot can generally be described by the Vogel-Fulcher-Tammann (VFT) equation [6–8]. For the strongly fragile cases, such as fluoride glass, the Cohen-Grest (CG) model provides a better simulation [5,9,10].

The compositional dependence is another factor to be considered. Many theories used to explain this dependence of glass properties are based on the additive models of Winkelmann and Schott [11,12]. This method assumes that a glass is an ideal mixture of components. Then, each property of glass, e.g., heat capacity and $T_{g}$, is expressed as a linear function of the compositional ratio.

Thus far, many attempts have been made to explain the correlation among viscosity, temperature, and composition in a specific family of glass, such as soda-lime glass. For example, Fluegel proposed equations in terms of the mole fraction of raw materials and their products [13]. Note that the inclusion of the product of the mole fractions enables us to describe the nonlinearity not included in the Schott model within the multiple linear regression method. Specifically, Fluegel derived three equations to predict the so-called isokom temperature (the temperature required to achieve a specific viscosity) of a specified chemical composition at three different viscosities (10^1.5^, 10^6.6^, and 10^12.0^ Pa·s) that are important in the manufacturing process of glass materials. Note that it is more convenient to predict the isokom temperature than the viscosity at a certain temperature because the temperature for a particular viscosity can vary over a wide range, depending on the chemical composition. One of the Fluegel equations is presented here,

(1) $$\begin{array}{rl} & T_{6.6}\left({\;}^{\circ }C\right)=939+5.81C_{Al_{2}O_{3}}-4.37C_{B_{2}O_{3}}\\ & \;\;\;\;-0.174{\left(C_{B_{2}O_{3}}\right)}^{2}+\ldots +0.320C_{B_{2}O_{3}}\\ & \;\;\;\;\times C_{Na_{2}O}+\ldots -0.034C_{Al2O_{3}}\times C_{B_{2}O_{3}}\\ & \;\;\;\;\times C_{Na_{2}O}+\ldots \end{array}$$

Here, $\;C_{Al_{2}O_{3}}$ represents the mole fraction (mol%) of Al_2_O_3_. This equation consists of 16 terms representing mole fractions of raw materials (excluding SiO_2_ as the remainder) and 22 terms representing the square and cubic terms, and the terms representing the products of the mole fractions of two and three raw materials. As the term selection and parameter optimization of the equation represent a type of machine learning, the Fluegel equation is valid only within a range of raw materials and their concentrations in which a sufficient number of data are available.

As the elemental composition is easy to prepare, several methods more sophisticated than the Fluegel equation have been proposed using elemental compositions. For example, Kim et al. extracted important descriptors from hundreds of thousands of candidates created by mathematically combining the band-gap energy, dielectric constant, and other properties of a target material to predict the dielectric breakdown [14]. However, we can hardly apply their method to the prediction of the isokom temperature, because obtaining the various physical and chemical properties of the glass materials is difficult and time consuming. Ghiringhelli et al. proposed a method for generating descriptors based on more basic features [15] – elemental properties such as ionization energy, electronegativity, and atomic radius. However, their method is also difficult to apply to the prediction of isokom temperatures, because glass materials usually consist of more than five elements; thus, the number of candidate features becomes too large in practice.

Considering the above situation, in this paper, we propose a new method for predicting the material properties for the following situations: (1) lack of information on the properties of the target material, (2) wide range of elements, or (3) properties of materials not limited to a particular family. The crucial point of our method is a novel descriptor called cation fingerprints. We have applied this method to glass materials, with the aim of predicting the isokom temperatures, one of the important viscosity properties in oxide glass materials, using our cation fingerprints combined with artificial neural networks.

This paper is organized as follows: In Section 2, we first explain the cation fingerprints, training data, and artificial neural networks that are used to predict the isokom temperatures. In Section 3, we present the results of the calculations. We discuss the properties of the cation fingerprints, which differentiate them from other descriptors, along with the optimization of the prediction models by the neural networks using all available data. Moreover, we discuss the performance dependence on hyperparameters for fingerprints (Section 3.1) and the accuracy of the experimental database (Section 3.2). Lastly, we present an attempt to predict the unexplored regions in Section 3.3. The accurate prediction in the unexplored region is important in finding novel materials but is not well predicted by the previous models. The conclusions are provided in Section 4.

## 2. Methodology

### 2.1. Cation fingerprints

To predict material properties using machine-learning techniques, it is necessary to convert the information of the material into descriptors. In the present study, we propose a new descriptor named ‘cation fingerprints’. Before explaining the cation fingerprints, we explain why a new descriptor is desired.

Descriptors can be roughly divided into two categories – those that can be evaluated only when the atomic structures of target materials are known and those that can be evaluated even when the atomic structures are unknown. Examples of the former are atomic coordinates, symmetry functions, smooth overlap of atomic positions, and graphs with atomic vertices from Voronoi tessellation [16–19]. Hence, we can employ some of these descriptors in platforms such as AFLOW-ML [20] and pymatgen [21] for our study.

The latter category is sometimes necessary because in practical experiments, we sometimes cannot control or even know the atomic structures of the material. In this situation, only the elemental compositions of the material or the ratio of the raw materials is known. Descriptors available in this case are very limited. The only information provided for machine learning is a chemical formula or elemental compositions. Mole fraction or the presence/absence of constituting elements or raw materials is a descriptor that is derived from chemical formula or elemental compositions in a straightforward manner. This descriptor is used in the Fluegel equation mentioned above. A severe drawback of this descriptor is that the number of terms increases rapidly with the number of elements increases. For example, when 30 raw materials are considered, there are more than 4000 ways to choose three materials. It is very difficult to determine which of the combinations is important without sufficient background knowledge and/or data. Ghiringhelli et al. proposed a descriptor using the basic elemental properties [15]. However, their descriptor is applicable only to binary systems or specific families.

Therefore, we propose a new descriptor. In developing this new descriptor, we were inspired by two recently proposed descriptors – the elemental-property-based attributes (we call ‘elemental attributes’ hereinafter) by Ward et al. [22] and the band-structure fingerprints by Isayev et al. [23]. Note that the elemental attributes are the largest among the four attributes available in magpie. To make the originality of our descriptor clear, we explain the two descriptors as follows: In evaluating the elemental attributes, first, data of 23 elemental properties (e.g., atomic number, electronegativity) are collected for each element from hydrogen to plutonium. Then, the average, minimum, maximum, variance, and mode of each property are calculated based on the elemental ratio of a target material. A total of 115 features are then obtained from the five statistical values for the 23 elemental properties. The band-structure fingerprint was generated from the simulation of the electronic band structure of the material. In this descriptor, the important energy range, such as ±10 eV from the Fermi level was divided into several bins. The number of bands was then included in the energy range of each bin at the target symmetry point in k-space. For example, considering the case of KNO_3_ (ICSD 384), there was one energy band between −10 and −7.5 eV, three bands between −7.5 and −5.0 eV, and no energy band between −5.0 and −2.5 eV. The fingerprints for the eight bins from −10 to +10 eV can be expressed as [1, 3, 0, 5, 0, 2, 0, 3]. The length of the entire fingerprint was the product of the number of symmetry points and bins.

Now, in terms of our cation fingerprints, because our target in the present study is oxide glass materials, we first collect the physical properties of the raw oxide materials [24,25]. Unlike the band-structure fingerprint, in which the high symmetry points are predetermined according to the crystal structure, the number of physical properties can be flexibly selected according to the purpose of the study. Specifically, we adopt density, enthalpy of formation, entropy of formation, melting point, and coordination number from the oxide, and ionic radius and electronegativity from its cation. Then, we can make seven frequency distribution tables from a given raw material composition for each of the seven properties.

**Figure 1.**
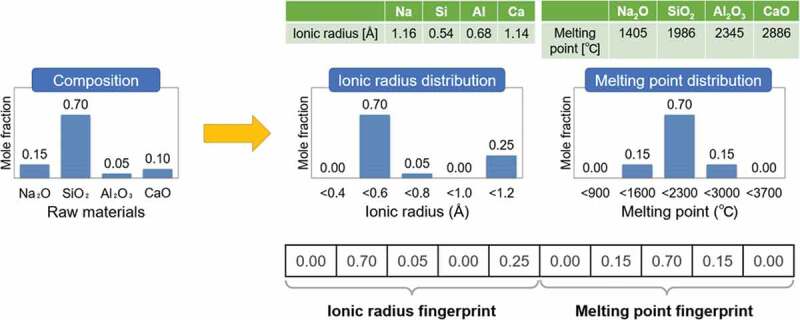
Schematic diagram of the generation of cation fingerprints.

Figure 1 shows an example of how to generate cation fingerprints of ionic radius and melting point for a simple soda-lime glass. For the cation ionic radius data, no elements correspond to the ionic radius between 0.2 Å and 0.4 Å. The only raw material with a cation ionic radius between 0.4 Å and 0.6 Å is SiO_2_, and its mole fraction is 70 mol%. The raw material with an ionic radius between 0.6 Å and 0.8 Å is 5 mol% of Al_2_O_3_, and no materials fit into the range between 0.8 Å and 1.0 Å. Finally, for an ionic radius between 1.0 Å and 1.2 Å, a total of 25 mol% of materials, from Na_2_O and CaO, belongs to this bin. Thus, the ionic radius fingerprint of [0.00, 0.70, 0.05, 0.00, 0.25] can be obtained from the frequency distribution of the five bins. To obtain the final fingerprints, the same procedure is repeated for all the physical properties we have. Note that the ranges of ionic radius and the melting point shown in Figure 1 are not realistic but are represented by simplified numerical values for clarity.

### 2.2. Training data

In the present study, we have developed a predictor for the isokom temperature of glass at three viscosity points of 10^1.5^, 10^6.6^, and 10^12.0^ Pa·s with the supervised machine-learning technique described in Section 2.3. Two databases are suitable for the purpose of training the predictor. One is SciGlass [26], which was used to select terms and optimize parameters in the Fluegel equations mentioned earlier. The other database is INTERGLAD [27], which was released earlier than SciGlass. It includes a regression analysis and glass design functions. Priven et al. claim that INTERGLAD is more suitable for commercial glass development owing to the 800,000 data records available on a variety of properties while SciGlass contains more detailed descriptions on synthesis procedures and original reference information [28]. However, both databases have recently been improved and there is no significant between them. Both have data about the material properties of more than 300,000 types of glass materials and have similar characteristics.

Note that the quality of data always presents an issue. Fluegel was conscious of this and used only calibrated data within a limited composition range to construct the Fluegel equation. An offset value was set for each reference (i.e., the data in the same reference have the same offset value), such that the same calibrated value would be obtained for the same compositions and conditions. This preprocessing increases the reliability of the model and eliminates the errors depending on experimenters. On the other hand, this approach cannot be applied to references that do not have enough data for mutual verification. Consequently, data of such references are discarded.

Because the purpose of this study is not the construction of a predictor with high accuracy but rather the verification of our novel approach, we used INTERGLAD without any preprocessing treatment or screening of data. In this study, data of raw oxide materials are also necessary to generate cation fingerprints. We collected data of seven properties (density, formation enthalpy, formation entropy, melting point, number of coordination, ion radius, and electronegativity) for 66 oxides and their cations from the materials handbook [29]. The number of isokom temperature data records for glass materials made from the combination of these 66 oxides is 1541, 12,103, and 5636 at the three viscosity points, respectively.

### 2.3. Multi-layer perceptron

For the model to predict via machine learning, we adopted the artificial neural network (ANN). This is one of the commonly used machine-learning techniques in nonparametric regression. In recent years, various studies have succeeded, using ANNs, in predicting complex situations that have never been accomplished before, such as image recognition, machine translation, and abstract strategy games [30–34]. For example, in materials science, ANN has been applied to the construction of interatomic potentials trained with first-principles calculations, construction of exchange-correlation functionals in density functional theory, and the prediction of the liquidus temperature of glass materials [16–18,35,36].

In this study, we used one of the simplest forms of ANN, a fully connected neural network called multi-layer perceptron. It has multiple hidden layers of neurons, and the layers are fully connected in a feed-forward way; that is, respective neurons are connected to all the neurons in the adjacent layers. In this study, we use 140 neurons in the input layer using cation fingerprints with 20 bins for the seven properties (20×7=140). The output layer has one neuron representing the isokom temperature in one of the target viscosities. The multiple hidden layers were optimized to have between 70 and 200 neurons as the input layer and to be inserted between the input and the output layers.

Theoretically, a neural network with a finite number of neurons in one hidden layer can emulate any continuous objective function [37,38]. In practice, however, two or more hidden layers are often needed to optimize network parameters. This is mainly because the back-propagation techniques used to minimize training errors cannot achieve the global minimum of errors. In this study, we used two hidden layers and confirmed that it provides sufficient performance. We used a rectified linear unit (ReLU) as the activation function. The ReLU typically learns faster and better than other activation functions in multi-layer neural networks [39]. To suppress the overfitting in the learning process, we adopted L2 regularization [40,41].

The network parameters were optimized using the adaptive moment estimation (Adam), which has been recently proposed and shown to be a fast algorithm for deep learning [42]. In this study, 20% of the total data were randomly selected and assigned in advance for the test set. After the machine-learning model trained from the remaining 80%, the model was evaluated using the prediction accuracy of the test set. All of the calculations were done using Keras with a Tensorflow backend [43,44].

## 3. Results

### 3.1. Performance dependence on hyperparameters for fingerprints

Before performing machine learning, it is necessary to set hyperparameters. That is, in the case of ANN models, we must determine the numbers of neurons and hidden layers, the type of activation function, etc. The settings of these hyperparameters have already been described in the previous section. In addition, in the case of machine learning via fingerprints, we have extra hyperparameters for the generation of fingerprints, that is, the choice of physical properties of raw materials and the number of bins (or the resolution of frequency distribution). These two hyperparameters are unique and critical only for the fingerprints. Thus, we examined the dependence of the machine-learning results on the two hyperparameters and set their values.

Table 1 shows the optimized results of the search space that was considered to find the best prediction model. Note that aside from the ANN model, we also examined four other models, the random forest, support vector machine, kernel ridge regression, and linear ridge regression, for comparison. The optimization details of these four models are listed in the supplementary material. In case all the data listed in the database are used, the well-tuned prediction models have small differences in prediction errors (neural network: 33.0°C, random forest: 33.1°C, support vector regression: 33.9°C, and kernel ridge regression: 34.2°C) except for the linear ridge regression (76.1°C). Note that the errors in this section are represented in terms of the root-mean-square (RMSE) of the randomly selected test sets.

**Table 1.** Hyperparameter tuning results. The optimization details of the other prediction models are listed in the supplementary material.

Hyperparameter	Search space	Optimized value
Prediction model	Neural network, random forest, support vector machine, kernel ridge regression, linear ridge regression	Neural network
Activation function	Sigmoid, Tanh, ReLU	ReLU
Number of hidden layers	1 to 6	2
Number of neurons in each hidden layer	70, 100, 140, 200	140
L2 regularization scale	0, 10^−5^ to 10^−2^	0.0001
Number of physical properties	1 to 7	7
Number of bins	5, 8, 10, 15, 18, 20, 24, 30, 50, 100	20

For the L2 optimal regularization scale, the optimal value was 0.0001, which provided a prediction error of 33.0°C. However, the improvement in the prediction accuracy was not significant compared to that of the 33.6°C without regularization. This result suggested that overfitting is unlikely to occur because of the sufficient number of training data.

The hyperparameters are listed in the last two lines of Table 1. The numbers of the physical properties and bins are hyperparameters specific to the cation fingerprints. We analyzed and examined the characteristics of the cation fingerprints in the following.

**Figure 2.**
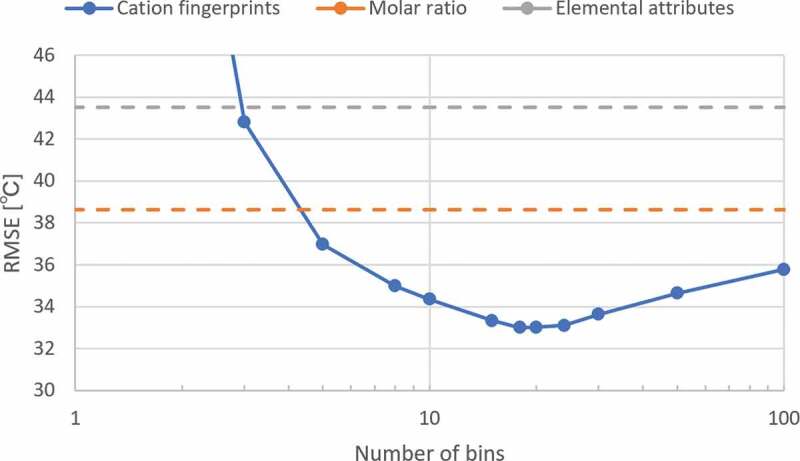
Prediction error of ANN models from different number of bins with seven physical properties. The two parallel dashed lines represent the prediction error from the elemental attributes and molar ratio, respectively.

Figure 2 shows the dependence of the prediction performance on the number of bins for the isokom temperatures of $log\left(\eta /\left(Pa\times s\right)\right)=6.6$. Two parallel dashed lines represent the test set errors of prediction using molar ratio and elemental attributes as descriptors, respectively. In the test of the other two descriptors, the best ANN structures were the same as the ANN structure optimized by the cation fingerprints. The line graph shows the test set error as a function of the number of bins, when the cation fingerprints are used as a descriptor. As the number of bins increases, the resolution of distributions becomes finer and the test set error decreases initially and then increases. As seen in Figure 2, 20 bins have the best prediction performance. Therefore, in this study, we decided to use 20 bins.

We speculated that in the limit of the number of bins, the prediction error of cation fingerprints converges to that of the molar ratio because of the following reason: Fingerprints are constructed by assigning a molar ratio of the raw material to each bin based on the distribution of the target raw property. In this process, as the number of bins increases, the average number of raw materials assigned to a bin decreases. Because the raw materials used in this study were 63 oxides, the average number of raw materials assigned to one bin was less than one in the cases of those that were larger than 63. As a result, the value of a bin is based on the molar ratio of one raw material, excluding the empty bins. Consequently, the prediction performance of the cation fingerprints and molar ratio was expected to be the same in the limit of the number of bins.

For the number and selection of physical properties, the optimization on them can not only improve the training accuracy but also provide clues to determine which physical properties are important. While the ANN is one of the black box models, we can use the nature of fingerprints to indirectly evaluate the importance of each physical properties by controlling the properties used for machine learning. First, we tested seven sets of fingerprints in which each property was removed from the seven properties, respectively. No significant differences in the test error are seen among the seven sets.

Next, we examined the cases of one or two properties. The diagonal and off-diagonal terms in Table 2 show the test errors (RMSE) of the models with the one-property and two-property fingerprints, respectively. In Table 2, the seven physical properties are denoted by capital letters, that is; A: electronegativity, B: coordination number, C: density, D: ionic radius, E: formation enthalpy, F: formation entropy, and G: melting point. Among the 28 combinations using the cation fingerprints in Table 2, the combinations of the oxide density and melting points explain the viscosity behavior with the smallest prediction error of 41.6°C. This is verifiable because a higher density or melting point of an oxide means that a higher temperature is required to achieve the same viscosity level. Using these analyses, the important physical properties explaining the isokom temperature can be estimated.

**Table 2.** Test set root mean squared error (°C) of ANN training from one (diagonal components) or two physical properties. Training was done by (a) cation fingerprints and (b) elemental attributes. Each physical property is indicated by one capital letter (A: Electronegativity, B: coordination number, C: density, D: ionic radius, E: formation enthalpy, F: formation entropy, and G: melting point).

**Figure 3.**
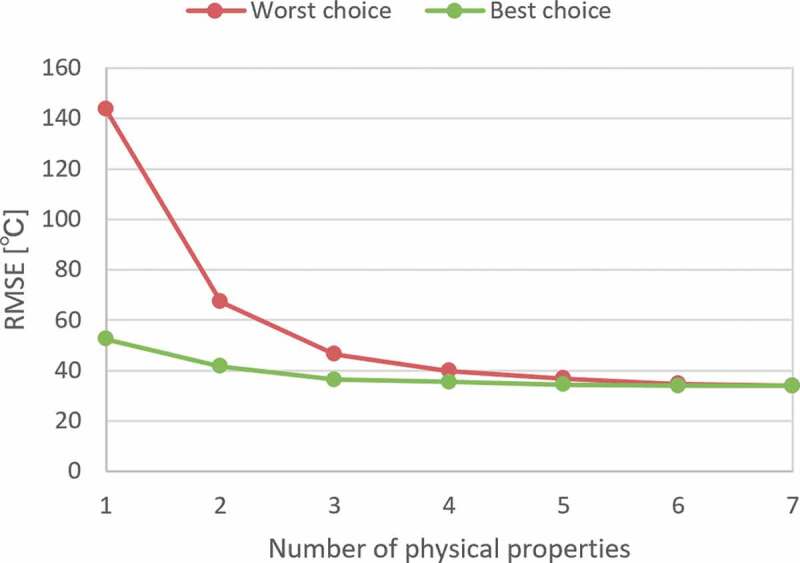
Test error of ANN training from fewer physical properties. Each line represents the worst and the best choices from each combination.

Figure 3 shows the dependence of the RMSE of the test prediction on the number of physical properties. The smallest and largest training errors within the same number of used properties, except for the case using all seven properties, are shown. As shown in the figure, the model becomes more stable and accurate when more physical properties are included. On the other hand, there was no significant decrease in the prediction performance despite some important properties missing. It can also be observed that there was no decrease in performance when relatively meaningless properties were added. Considering the above, we decided to generate cation fingerprints from frequency distributions of 20 bins for the seven physical properties of raw oxides for the analyses in Sections 3.3 and 3.4.

### 3.2. Comparison with elemental attributes

We can perform an evaluation similar to Table 2 for the elemental attributes because the properties used to generate descriptors can be chosen. The results of the same analysis for the attributes are shown in Table 2. Although the order of the RMSE among the 28 combinations was not exactly the same between Table 2, the isokom temperature was most accurately predicted when the oxide density or cation ionic radius was used with the oxide melting point in both cases.

However, a distinct difference was observed between the cation fingerprints and elemental attributes. In the prediction by cation fingerprints using only one physical property, the melting point (G) provided the lowest prediction error, whereas it provided the largest error in the prediction by elemental attributes using one physical property. This difference can be attributed to the fact that the fingerprint contains richer information than that of the set of statistical quantities used in the elemental attributes such as mean and variance.

In addition, in cases where two and seven properties were used, the prediction accuracy of the cation fingerprints was higher than that of the elemental attributes. From these results, it can be concluded that the cation fingerprints were superior to the elemental attributes in both the prediction performance and explanation capabilities.

### 3.3. Accuracy of the experimental database

In the previous Subsection, we showed that the cation fingerprints were better descriptors than that of the molar concentration. However, the prediction error of the cation fingerprints, which was approximately 33°C, was larger than that of the Fluegel equation, which was approximately 10°C. This was because we did not restrict the range of raw concentrations and treated both silicate and non-silicate glasses with a single prediction model. However, the prediction error difference was still large despite the points previously mentioned. We suspected that one possible reason was the effect of the data screening. Thus, we analyzed the data using the linear regression of the molar concentrations for a more equitable comparison.

As mentioned previously, Fluegel preprocessed data. References are screened, experimental results are shifted depending on original references. As it is difficult to perform the Fluegel screening method, we performed screening using a purely data-driven method that does not require any background knowledge of data. For this purpose, we adopted random sample consensus (RANSAC) [45–47], which is a robust regression method against outliers and was originally suggested as an algorithm for image analysis. We describe the details of RANSAC analysis and their results in the supplementary material.

In our results, when the RANSAC was filtered out at approximately half of the data, the prediction errors became similar to those of Fluegel. The RANSAC screening was observed to be helpful for the robust prediction under noisy conditions because some experimental results would have been removed by the RANSAC. For example, the experimental data with the same composition, but with a temperature difference of greater than 50°C, which Fluegel regarded as irrelevant, would have been removed. We can also say that the large difference in the RMSE between our results and those of Fluegel was possibly due to whether the preprocessing and data screening was performed. However, we must be careful when using the RANSAC screening for prediction. This is because in this screening, valuable data can be lost when the amount of inlier and error threshold is assumed in advance. It remains a future task to investigate the validity of filtering out noisy experimental data in an experiment-based database. Because it is difficult to apply the same preprocessing to all the data in the databases, and our goal is the evaluation of fingerprints as a generally usable descriptor, all machine learning in this study was performed without preprocessing the data.

### 3.4. Fingerprints as descriptors for untrained novel materials

To demonstrate the advantage of the fingerprints, we tested a virtual extreme case of material search, which cannot be performed at all by the mole fractions. For this purpose, we set a specific target oxide in raw materials, and trained the ANN model using materials that do not contain the target oxide. Then, we tested the prediction accuracy of the glass materials, including the target oxide with the ANN model trained in this manner.

We searched the entire database for raw materials in which the percentage of glass containing those raw materials is between 10% and 30%. This range was determined based on the 20% random test set we examined earlier. This range allows us to conduct as many test sets as possible while making it easier to compare results with the 20% random test set. In other words, we have to consider the trade-off between the small test set, which may not properly reflect raw material dependency, and the significant change in training size, which may influence ANN model performance. Table 3 shows the nine oxides that satisfy the above condition. In the case of SnO_2_ for example, 1307 glass materials contain SnO_2_, but the remaining 10,796 do not contain SnO_2_ at all. Then, the ANN model was trained by the 10,796 data points, and the isokom temperatures of 1307 glass materials including SnO_2_ were predicted.

**Table 3.** Nine target materials with their respective test set size and training set size.

Raw materials	MgO	Li_2_O	ZrO_2_	PbO	SrO	P_2_O_5_	TiO_2_	Sb_2_O_3_	SnO_2_
Test set size	3631	3044	2673	2593	2518	2036	1895	1775	1307
Training set size	8472	9059	9430	9510	9585	10,067	10,208	10,328	10,796

**Figure 4.**
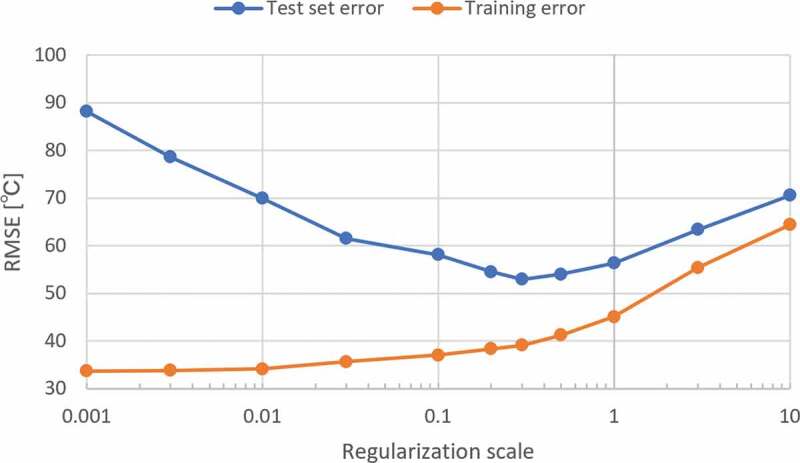
Penalty dependence of averaged root mean squared error of the nine test sets fromTable 4.

The prediction error was large when we used the model settings discussed in Subsection 3.1. Hence, we re-optimized the prediction model and investigated the requirements to explore novel combinations of materials which have not been examined yet. In Figure 4, we show the dependence of the averaged error over the nine test sets in Table 4 on the L2 regularization scale. The minimum prediction error is obtained when the regularization scale is 0.3. Although the final prediction error, 53°C, is higher than the random prediction error of 39°C, the result is meaningful because the material property is predicted from the training data without including the target raw materials. In addition, the prediction errors of elemental attributes and molar concentrations under the same situation are 73°C and 62°C, respectively. That is, cation fingerprints show better performance than the other descriptors.

**Table 4.** Root mean squared error (RMSE) of test sets with various L2 regularization scales.

Raw materials	MgO	Li_2_O	ZrO_2_	PbO	SrO	P_2_O_5_	TiO_2_	Sb_2_O_3_	SnO_2_	Average
RMSE of test set (°C) with L2 = 0.0001	63.06	92.87	53.32	148.43	56.69	453.72	62.14	73.58	31.23	115.00
RMSE of test set (°C) with L2 = 0.001	57.92	80.36	52.67	129.28	55.13	271.85	52.94	62.45	30.56	88.13
RMSE of test set (°C) with L2 = 0.01	51.71	76.77	52.56	100.73	54.20	168.40	48.71	45.73	30.55	69.93
RMSE of test set (°C) with L2 = 0.3	48.68	64.50	48.61	60.89	54.81	81.91	47.34	39.74	30.27	52.97
RMSE of test set (°C) with L2 = 1.0	53.33	66.21	50.93	86.53	58.00	80.52	47.16	39.15	37.20	57.67

We attempted to determine in more detail how a prediction is possible in this extreme situation. As we can see from Figure 4, with the regularization scale, the training error continuously increases, whereas the test error decreases gradually until the regularization scale reaches 0.3, then slowly increases. This is typical behavior of the phenomena known as the tradeoff between overfitting and underfitting. Note that the value of the regularization scale of 0.3 at the test error minimum was higher than that of the optimized scale at 0.0001. This was conducted by randomly dividing the data sets. This may suggest that the present attempt is a situation where overfitting is likely to happen and is different from normal training and prediction. We also examined the effect of the number of bins, and found that the optimal number of bins was 20, which was the same as in Subsection 3.1 from the randomly divided data sets (for details, see Figure S1 in supplementary materials).

In addition, we compared the performance of ANN with the other four prediction models as in Subsection 3.1 (for details including the hyperparameter setting, see Table S1 in supplementary materials). The results of the prediction errors for the random forest, support vector regression, kernel ridge regression, and linear ridge regression were 61.7°C, 57.7°C, 58.91°C, and 77.0°C, respectively. The prediction errors significantly increased in all models including the ANN model. Compared to the case in Subsection 3.1, the difference in the predictive accuracy between the ANN and other models was slightly larger. In both cases, the ANN model had the best predictive performance. Therefore, it can be concluded that the ANN model fits well with the cation fingerprints for the prediction of material properties.

In Table 4, we show the dependence of prediction errors of respective target oxides and their average on the regularization scale. While some oxides (SnO_2_, ZrO_2_) are well predicted even without regularization, some others (P_2_O_5_, Li_2_O) are poorly predicted, even with a large regularization scale. In particular, the error in the case of P_2_O_5_ is the largest. This large error may be attributed to the fact that phosphorous is a nonmetallic element and oxides of nonmetallic elements are rarely contained in this database, other than phosphorus pentoxide. Specifically, only 163 data records of SO_3_ and 7 of SeO_2_ are available as oxides of the nonmetallic elements.

**Figure 5.**
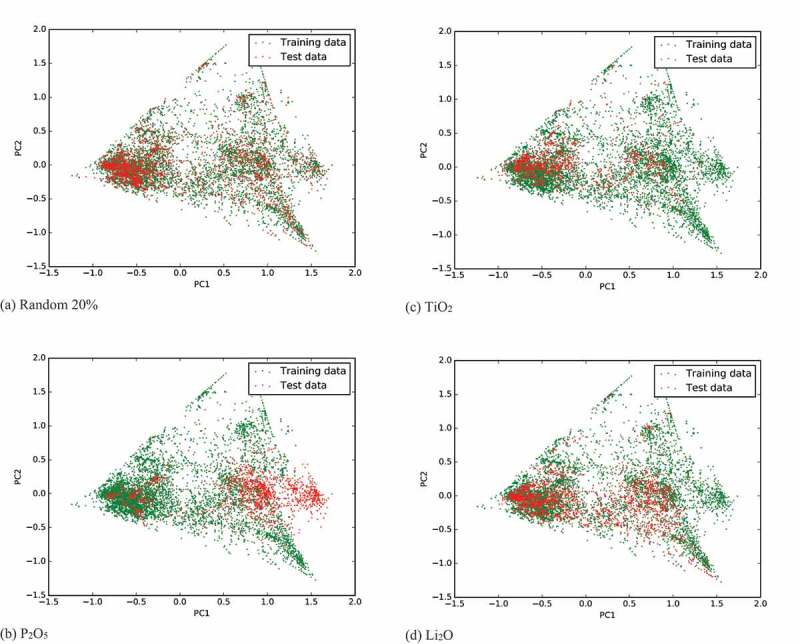
Distribution of first two principal components of fingerprints. Test sets are divided by (a) randomly selected 20%, (b) P_2_O_5_ test case, (c) TiO_2_ test case, and (d) Li_2_O test case.

We performed principal component analysis (PCA) [48,49] to investigate the cause of this error. Figure 5 represents the distributions of the 1st and 2nd principal components in the case of (a) randomly divided test set, (b) P_2_O_5_ test set, (c) TiO_2_ test set, and (d) Li_2_O test set, respectively. As seen in Figure 5(b), in the case of the P_2_O_5_ test, many test materials (containing P_2_O_5_) are distributed in the right-hand area where no training data exist. However, in the case of Li_2_O, which has the second largest prediction error, most of the test data are distributed in the region where the training data exist. A similar feature is seen in the TiO_2_ case, which has a moderate prediction error. This PCA analysis reveals why the prediction error of a test set is large in the case of P_2_O_5_. We can also understand why the material property can be predicted from the training data without including the target raw materials. However, we cannot determine why the error is the second largest in the case of Li_2_O from this analysis. This may be because in the present PCA analysis, the dimension of fingerprints is reduced from 140 (seven properties by 20 bins) to 2, which may cause the loss of some information important to Li_2_O.

Considering the last speculation in the previous paragraph, we tried another analysis, which is based on the cosine similarity. Cosine similarity measures the similarity not of the lengths but of the directions of the two vectors via the inner product. It is widely used in high-dimensional data processing, such as text matching and data mining [50,51]. In our research, a fingerprint is regarded as a 140-dimensional vector. Because this vector constantly has an identical length (specifically, the length measured by the L_1_ metric) because the sum of the concentrations was constantly 100%, the cosine similarity method, which is specialized for angular information, was expected to be effective. The cosine value of two fingerprint vectors is defined as the cosine of the angle between the two vectors. Note that two fingerprints are more similar as the cosine value approaches 1.0. We also define the maximum cosine similarity – the maximum of the cosine value between the fingerprints of a material in the test set and that of a material in the training set.

**Figure 6.**
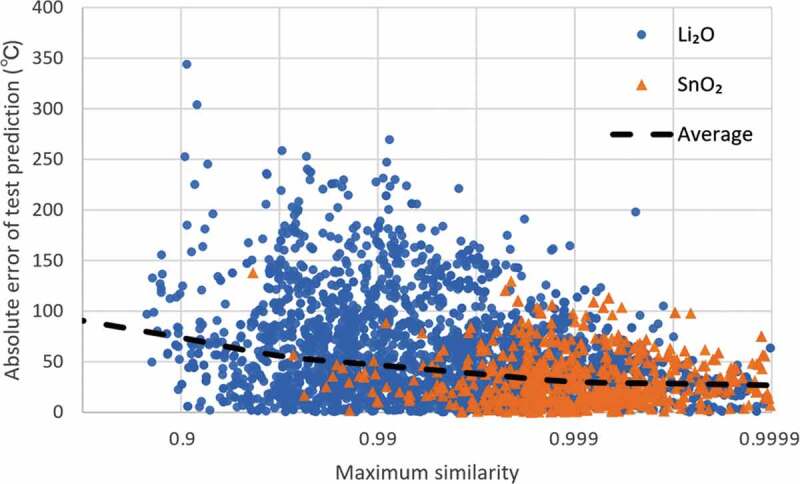
Maximum cosine similarity for training data (e.g. one dot of 0.99 means that this test data has no training data with cosine similarity larger than 0.99.) versus absolute error in Li_2_O and SnO_2_ test sets from Table 4. Dashed line represents the changes in mean absolute error over similarity range.

In Figure 6, we plotted the reverse logarithm scale of the maximum cosine similarity versus prediction errors. The blue circles and orange triangles represent the Li_2_O and SnO_2_ test sets (test sets with the smallest prediction error), respectively. The dashed line represents the mean absolute error (MAE) within half the reverse logarithm magnitude (e.g., from 0.900 to 0.968 and from 0.968 to 0.990) of the maximum cosine similarity range. The MAE is 74°C for a maximum cosine similarity of 0.9, whereas it is 27°C for a similarity of 0.9999. An inverse correlation between the cosine similarity and prediction error was evidently observed – the closer the fingerprint of the test material is to one of the trained fingerprints, the more accurate the prediction.

We found that the tendency between the similarity and accuracy was the same for the cosine similarity analysis results using the molar concentration and elemental attributes (see Fig. S2 in the supplementary material). This means that the prediction results from any of the descriptors tend to be more accurate when there are similar materials in the training data. However, the three descriptors used in this study showed different scales of cosine similarities. For example, the required values of similarity with a mean absolute error of less than 50°C were about 0.984, 0.9994, and 0.9999996 for cation fingerprints, molar concentration, and elemental attributes, respectively. Therefore, the absolute criterion will depend on the combination of the target material property and descriptor.

From these results, we can say that the properties of a material are relatively predictable when one of the ‘input features’ in the training set is similar to that of the test material. Furthermore, it is possible to evaluate the prediction accuracy quantitatively using cosine similarity, while the required degree of similarity seems to be vary depending on the descriptors. However, even the similarity analysis could not reveal the reason why the cation fingerprint is more robust to the extreme case of material search than other descriptors. To clarify this remains as a future task.

Finally, we show the experimental and predicted isokom temperatures at a viscosity level of 10^6.6^ Pa·s and raw compositions in Table 5. The first line shows the data of a test material with high Li concentration taken from the Li_2_O test set, and the others show those of training materials that have the most similar fingerprints in the training set. In this case, the regularization scale was set to 0.3, as the maximum similarity is 0.9916. One interesting point seen in Table 5 is that all of the three training materials contain oxides not included in the test material, that is, CaO, La_2_O_3_, and TiO_2_. This may be related to the fact that Li has an oxide density similar to Si and Ca, and a similar oxide melting point to those of Si and Ti, along with similar electronegativity to those of Ca and La. It is interesting to note that the isokom temperatures of the top-three materials with high similarity are quite different from that of the test material, even though the isokom temperatures of all of them are well predicted.

**Table 5.** One example of Li_2_O test material and the top three training materials with similar fingerprints.

		Raw oxides composition (mol %)								Isokom temperature at 10^6.6^ Pa·s	
	Fingerprint similarity	SiO_2_	Al_2_O_3_	PbO	As_2_O_3_	CaO	La_2_O_3_	TiO_2_	Li_2_O	Experimental value	Predicted value
Test material		70.77	14.91	1.88	0.10				12.34	944	980.4
Training materials	0.9916	81.33	13.69	2.50		2.48				760	741.1
	0.9906	76.63	13.89	3.17		6.31				740	732.3
	0.9900	82	12				4	2		1110	1087.5

In the above, we demonstrate the successful performance of our method. However, we would like to note that this does not mean success in extrapolation; extrapolation is still challenging because we have high prediction error in the cases without materials that have high cosine similarity to the test material in the training ones. Instead, our results show that some of the extrapolation situations can be successfully transformed into interpolation situations. In our case, this is confirmed from the cosine similarity of descriptors. In addition, we show that it is possible to estimate the prediction accuracy by evaluating the descriptor similarity. However, further research is needed to determine how much the similarity penalized correlations can be generalized.

## 4. Conclusions

We proposed a novel material descriptor named cation fingerprints for property prediction and material search. The cation fingerprint can be generated based on the concentrations of raw materials and the frequency distribution of several simple properties of raw materials. This does not require any basic knowledge of target material; rather, it uses the simple properties of the raw material, such as density, strength, and atomic configuration. We demonstrated the effectiveness of the cation fingerprints through the prediction of the viscosity properties of oxide glass materials via an artificial neural network (ANN) model. While under limited concentration range with a multiple linear regression of molar concentration yielding a root-mean-square prediction error of 47.4°C, our cation fingerprints with the ANN model give prediction error of 33.0°C without any concentration limit. We also investigated how much of the existing data is useful as we explore uncharted materials. We assumed an extreme condition for the materials search in which the most unique raw materials ever tested were examined. A test that excludes certain raw materials from the model training has shown that fingerprints with the ANN model can predict the effects of novel raw materials that ANN model has never seen with an error of 53.0°C, which is better than the other descriptors examined in this study, elemental attributes (73°C) and molar concentrations (62°C). Based on these results, it can be concluded that the cation fingerprints proposed in this work were effective in the prediction and exploration of material properties. In addition, we suggest that the maximum cosine similarity of the descriptors in the test material and materials in a training set can be used to estimate the prediction accuracy.

## Supplementary Material

Supplemental Material
