# Supplementary material for: Prediction of viscosity behavior in oxide glass materials using cation fingerprints with artificial neural networks
Source: Sci Technol Adv Mater. 2020 Jul 22;21(1):492–504. doi: 10.1080/14686996.2020.1786856 (PMC7476533; doi:10.1080/14686996.2020.1786856)
Supplement: Supplemental Material [file TSTA_A_1786856_SM9149.pdf]

Supplementary Information

Prediction of viscosity behavior in oxide glass materials using cation fingerprints with artificial neural networks

Jaekyun Hwang[a*], Yuta Tanaka[b] , Seiichiro Ishino[c] , and Satoshi Watanabe[a, d*]

[a]Department of Materials Engineering, The University of Tokyo, Tokyo 113-8656, Japan; [b]Department of Physics, The University of Tokyo, Tokyo 113-0033, Japan (Present address: Nippon Steel Corporation, Chiba 293-8511, Japan); [c]Institute of Industrial Science, The University of Tokyo, Tokyo 153-8505, Japan (Present address: NISSAN ARC, LTD. Kanagawa 237-0061, Japan); [d]Center for Materials Research by Information Integration, Research and Services Division of Materials Data and Integrated System, National Institute for Materials Science, Tsukuba 305-0044, Japan

e-mail for the *corresponding authors: hwang@cello.t.u-tokyo.ac.jp, watanabe@cello.t.u-tokyo.ac.jp

Prediction models	Optimized hyperparameters	Root mean squared error (°C) of Isokom temperature prediction
Neural network	Number of hidden layers = 2<br>Number of neurons in each hidden layer = 140<br>Activation function = Rectified linear unit<br>Regularization scale = 0.0001	32.95
Random forest	Number of trees = 100<br>Maximum number of features = 56 (40%)<br>Maximum depth of tree = 50	31.40
Support vector regression	Kernel = Radial basis function<br>Cost function = 2000<br>γ = 5.0	33.86
Kernel ridge regression	Kernel = Radial basis function<br>Regularization scale = 0.0001<br>γ = 1.0	34.17
Linear ridge regression	Regularization scale = 0.0001	76.06

Table S1. Optimized hyperparameters and prediction accuracy of five regression models considered in this study (in Section 3.1). Training of prediction models other than the neural network was done by Scikit-learn [1].

In this study, we tried to predict the isokom temperature of glass materials using a neural network. We also investigated the prediction results obtained using other prediction models shown in Table S1. We tested four prediction models provided by the Scikit-learn package. The brief introduction of each model is as follows.

Linear ridge regression (LRR) is a model that applies L2 regularization to the training of linear regression. So the structure of the trained model has the same shape as the linear regression. Kernel ridge regression (KRR) is one of the nonlinear regression models that estimate conditional expectation using the kernel function [2]. This model applies regularization to the optimization of

kernel functions. Support vector regression machine (SVR) is a model consisting of a set of hyperplanes [3]. In this study, the kernel function was used to minimize the margins of the hyperplanes fast. Random forest (RF) is a model that consist of an ensemble of decision trees [4,5]. Ensemble gives more flexible and accurate prediction results than a single decision tree.

Each hyperparameter for the prediction models was optimized in the same way as the ANN model. Table S1 shows the optimized hyperparameter settings for respective models and the corresponding prediction errors.

		Fluegel's paper [6]			This study		
Viscosity level [log $(\eta/Pa\ s)$]		1.5	6.6	12.0	1.5	6.6	12.0
Linear regression without data preprocessing	Total number of data				1145	3446	1328
	Prediction R^2 score				0.8580	0.8463	0.8346
	Prediction RMSE (°C)				42.2437	47.3623	51.9269
Linear regression with data preprocessing	Number of inliers	1090	640	597	782	1776	652
	Inlier R^2 score	0.9881	0.9812	0.9835	0.9888	0.9917	0.9846
	Inlier RMSE (°C)	16.5334	9.7859	8.8174	9.8612	10.2852	12.0561

Table S2. Total data and R^2 scores from Fluegel's paper (with data offset) and from the dataset in this research without and with data preprocessing (RANSAC).

We compare the prediction performance between our model and the Fluegel equation. As mentioned in the main text, Fluegel preprocessed data. Because we have a different database and different data references from those Fluegel used, we compared the representation of the Fluegel equation using different data. Table S2 shows our training results of linear regressions obtained with exactly the same terms suggested by Fluegel, together with the original Fluegel's results [6]. We did not preprocess the data, while the composition of data was limited to the same range as the Fluegel's data. The accuracy of predictions is evaluated by R^2 score, i.e., coefficient of determination. The total number of data records is 2.5 times larger than that of Fluegel's data. The prediction accuracy is lower than that of the Fluegel equation, as expected.

Random sample consensus (RANSAC) iteratively generates regression models from randomly selected data subsets. At each step, we evaluate the prediction errors and count the number of inliers with error smaller than a predetermined threshold value. Then, we update the model when it has more inliers than the model at the previous step. In the right lower part of Table S2, we show the results obtained using RANSAC with one of the typical outlier thresholds, $N^{-1/5} \times MAD$, where N is the number of data records and MAD is the median absolute deviation [7,8].

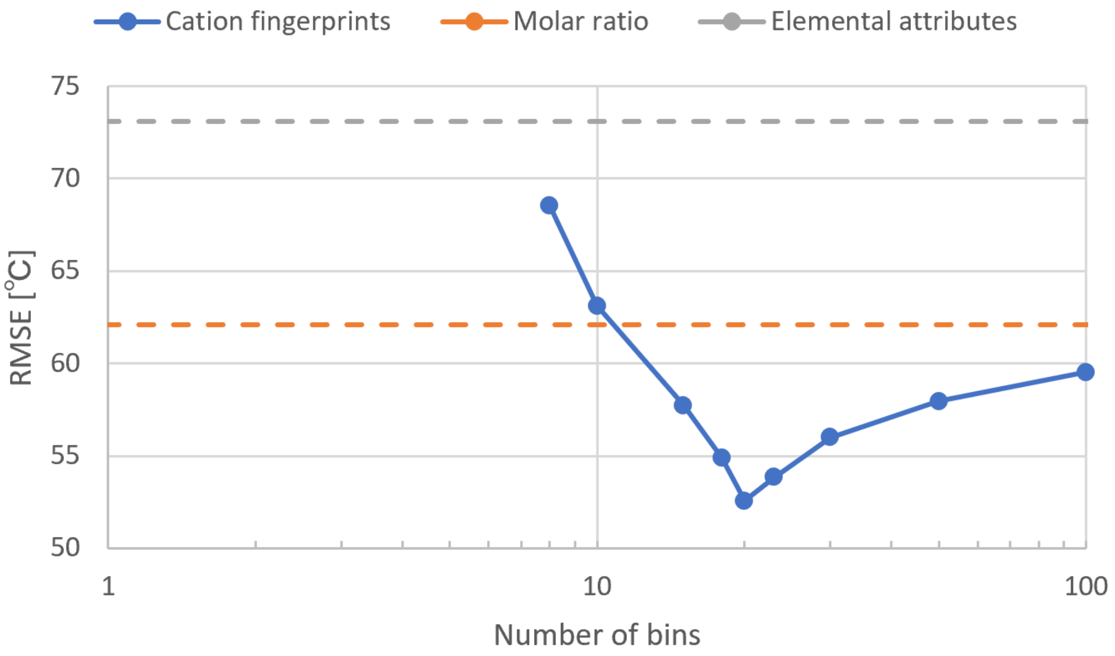

Figure S1. Dependence of prediction error of ANN models on the number of bins. Two parallel dashed lines represent the prediction errors from elemental attributes set and molar ratio, respectively.

In the extreme case of prediction in Subsection 3.4, we examined the dependency on the number of bins, one of the hyperparameters of the cation fingerprints. The results are shown in Figure S1. The lowest prediction error was obtained when the same number of bins (20 bins) as in the case of Figure 2 in the main manuscript was used. The variation of the prediction error with the number of bins is wider than that in Figure 2, and the sharp dip appears at 20 bins.

Although there is a difference in the shape of the graph as mentioned above, two characteristics seen in Figure 2 are also seen in Figure S1. First, the prediction error increases when too many bins are used. Second, the prediction error seems to converge to the prediction error of the molar ratio in the limit of large number of bins.

Prediction models	Optimized hyperparameters	Root mean squared error (°C) of Isokom temperature prediction
Neural network	Number of hidden layers = 2<br>Number of neurons in each hidden layer = 140<br>Activation function = Rectified linear unit<br>Regularization scale = 0.3	52.56
Random forest	Number of trees = 100<br>Maximum number of features = 28 (20%)<br>Maximum depth of tree = 30	61.69
Support vector regression	Kernel = Radial basis function<br>Cost function = 700<br>$\gamma = 0.5$	57.74
Kernel ridge regression	Kernel = Radial basis function<br>Regularization scale = 0.2<br>$\gamma = 0.2$	58.91
Linear ridge regression	Regularization scale = 1.0	77.01

Table S3. Optimized hyperparameters and prediction accuracy of five regression models considered in this study (in Subsection 3.4). Training of the prediction models other than the neural network was done by Scikit-learn [1].

Table S3 shows the hyperparameter settings and prediction results of five prediction models under the extreme prediction situations in Subsection 3.4. Hyperparameters of each model shown in Table S3 were re-optimized by the same way as the ANN model in Subsection 3.4. Prediction errors have increased significantly in all five models, including the ANN model. The prediction performance difference between the four models, excluding LRR, was not noticeable in Table S1 (31.4 °C - 34.2 °C) but a little wider in Table S3 (52.6 °C - 61.7 °C). In both cases, the ANN model shows a good predictive performance.

Note that the hyperparameters of all models have changed to prevent overfitting. Compared with

Table S1, the increase in optimal regularization scales is seen in all models using a regularization scheme. In SVR and KRR, which are prediction models using kernel trick, the optimal value of gamma decreases. Since it is known that the decrease of the gamma value tends to suppress overfitting, the decrease of gamma value seen in Table S3 can have a similar effect as the increase of the regularization scale. The cost function, another hyperparameter of the SVR, is a hyperparameter that determines how strictly the outlier is handled to set margins. The decrease of this hyperparameter from 2000 to 700 makes the prediction model more robust to outliers. Finally, in the RF, the maximum number of features decreases from 56 (40%) to 28 (20%), and the maximum depth of tree from 50 to 30. These changes can suppress overfitting by lowering the correlation among each decision tree and increasing the predictive power of the entire forest.

(a)

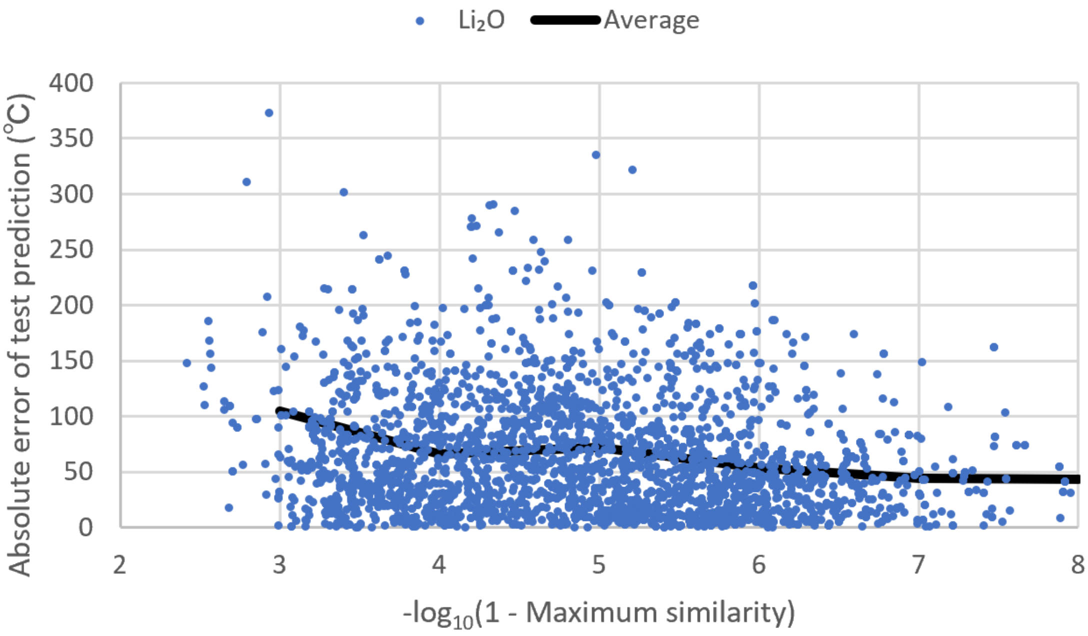

(b)

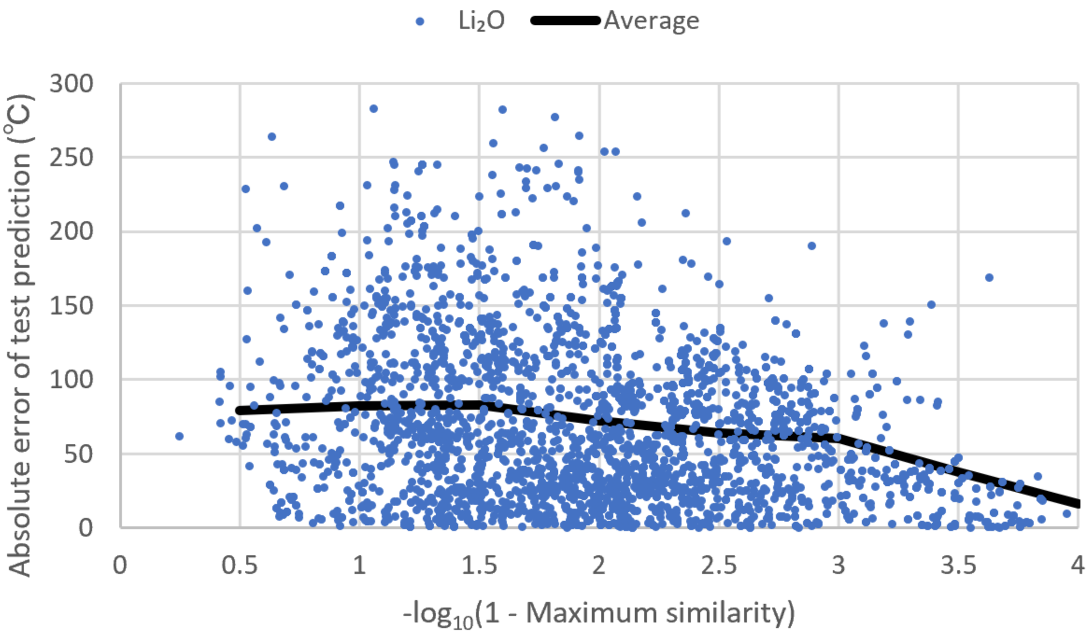

Figure S2. Maximum cosine similarity for training data versus absolute prediction error from (a) elemental attributes and (b) molar concentrations.

Figure S2 plots the relationship between maximum cosine similarity and the prediction error for the training results in the cases of (a) elemental attributes and (b) molar concentrations. Each dot represents a test material, and the solid black line represents the mean absolute error. It can be seen that the prediction accuracy is high if similar materials are included in the training set as in the case of fingerprints. Since the same pattern is seen in the three descriptors, this trend seen between similarity and predictability may be in common in machine learning using compositional descriptors.

However, similarity-accuracy distribution pattern or similarity scales required to secure target accuracy are all different. If we want a mean absolute error to be below 50°C in the prediction, maximum cosine similarity of 0.984, 0.9994, 0.9999996, or higher is required in cation fingerprints, elemental attributes, and molar concentrations, respectively.

References

1. Pedregosa F, Varoquaux G, Gramfort A, et al. Scikit-learn: Machine learning in Python. the Journal of machine Learning research. 2011;12:2825-2830.
2. Saunders C, Gammerman A, Vovk V. Ridge regression learning algorithm in dual variables. 1998.
3. Drucker H, Burges CJ, Kaufman L, et al., editors. Support vector regression machines. Advances in neural information processing systems; 1997.
4. Breiman L. Random forests. Machine learning. 2001;45(1):5-32.
5. Geurts P, Ernst D, Wehenkel L. Extremely randomized trees. Machine learning. 2006;63(1):3-42.
6. Fluegel A. Glass viscosity calculation based on a global statistical modelling approach. Glass Technology-European Journal of Glass Science and Technology Part A. 2007 Feb;48(1):13-30.
7. Choi S, Kim T, Yu W. Performance evaluation of RANSAC family. Journal of Computer Vision. 1997;24(3):271-300.
8. Subbarao R, Meer P, editors. Heteroscedastic projection based M-estimators. 2005 IEEE Computer Society Conference on Computer Vision and Pattern Recognition (CVPR'05)-Workshops; 2005: IEEE.
